# Hyperexpressed Netrin-1 Promoted Neural Stem Cells Migration in Mice after Focal Cerebral Ischemia

**DOI:** 10.3389/fncel.2016.00223

**Published:** 2016-09-30

**Authors:** Haiyan Lu, Xiaoyan Song, Feng Wang, Guodong Wang, Yuncheng Wu, Qiaoshu Wang, Yongting Wang, Guo-Yuan Yang, Zhijun Zhang

**Affiliations:** ^1^Department of Neurology, Shanghai General Hospital, Shanghai JiaoTong UniversityShanghai, China; ^2^Neuroscience and Neuroengineering Research Center, Med-X Research Institute, Shanghai Jiao Tong UniversityShanghai, China

**Keywords:** adeno-associated virus, ischemia, mice, netrin-1, neural stem cell

## Abstract

Endogenous Netrin-1 (NT-1) protein was significantly increased after cerebral ischemia, which may participate in the repair after transient cerebral ischemic injury. In this work, we explored whether NT-1 can be steadily overexpressed by adeno-associated virus (AAV) and the exogenous NT-1 can promote neural stem cells migration from the subventricular zone (SVZ) region after cerebral ischemia. Adult CD-1 mice were injected stereotacticly with AAV carrying NT-1 gene (AAV-NT-1). Mice underwent 60 min of middle cerebral artery (MCA) occlusion 1 week after injection. We found that NT-1 mainly expressed in neuron and astrocyte, and the expression level of NT-1 significantly increased 1 week after AAV-NT-1 gene transfer and lasted for 28 days, even after transient middle cerebral artery occlusion (tMCAO) as well (*p* < 0.05). Immunohistochemistry results showed that the number of neural stem cells was greatly increased in the SVZ region of AAV-NT-1-transduced mice compared with control mice. Our study showed that overexpressed NT-1 promoted neural stem cells migration from SVZ. This result suggested that NT-1 is a promising factor for repairing and remodeling after focal cerebral ischemia.

## Introduction

Cerebral blood flow disorder causes a series of reactions after stroke, which results in the death of neurons and nerve function damage (del Zoppo and Mabuchi, 2003). Neurogenesis occurs for a lifetime in rodents or in humans (Eriksson et al., 1998; van Praag et al., 1999). Although endogenous neurogenesis is activated after injury, it is insufficient to repair the damaged area of brain by itself in animal models (Göritz and Frisén, 2012). Neurogenesis enhanced by exogenous factor have been related with improved functional recovery in many studies (Hermann and Chopp, 2012). The role of neurogenesis in the early stage of ischemia is probably due to the release of growth factors, but not due to neuronal replacement (Chen et al., 2003). Integration of newborn cells into the existing local neural circuits may improve neural functions in the delay phase of stroke (Nakatomi et al., 2002). Netrin-1 (NT-1), an axon guidance factor, plays an important role in the axon guidance and cell migration in the development of nervous system (Hedgecock et al., 1990). The NT-1 receptor, neogenin is a key regulator of adult neurogenesis (O’Leary et al., 2015). The expression of endogenous NT-1 protein was significantly increased after cerebral ischemia compared with the sham mice (Tsuchiya et al., 2007). Endogenous NT-1 may participate in the repair after transient cerebral ischemic injury, and this repair is not enough to compensate the cerebral ischemic injury. Additional exogenous NT-1 in mouse brain attenuates apoptosis, promotes angiogenesis, white matter repairing and remodeling after focal cerebral ischemia (Lu et al., 2012; He et al., 2013; Liao et al., 2013). However, appropriate strategies were needed to make NT-1 stable expression for promoting stroke recovery.

Neural stem cell is the kind of cells with the capacity of proliferation, self-renewal and multi-differentiation. It can produce various kinds of neural cells by asymmetric cell division after injury in adult (Sommer and Rao, 2002; Barkho and Zhao, 2011). Neural crest stem cells and central nervous system (CNS) stem cells were classified according to its distributions (Kennea and Mehmet, 2002). Nestin expressed in the early stage of embryonic neural epithelium and disappeared after birth, and was considered to be one of the markers of neural precursor cells in the development of CNS (Gilyarov, 2008). Recent study demonstrated that Nestin not only exists of all mammalian embryonic development stages, but also widely distributed in adult CNS (Xu R. et al., 2010). Nestin mainly expressed in the activated precursor cells in the CNS and peripheral nervous system. Nestin expression decreased in the mature nervous system while re-increased in the damaged nervous system; its expression level was positive correlated with neurological functional recovery after injury (Krupkova et al., 2010).

Recent study had showed that gene transfer could be a promising approach for cerebrovascular diseases (Gan et al., 2013). *In vivo* non-virus gene delivery is relatively safer than virus-mediated delivery but is limited by its inefficiency (Kamimura et al., 2011). Target gene effective delivery and stable expression are needed. Recombinant adeno-associated virus (rAAV) is a new carrier in the field of gene therapy in recent years (Palomeque et al., 2007). Compared with other vectors, adeno-associated viral vector is more suitable as a vector for gene therapy because it has the following characteristics: (1) it is more safety, mild immune response, less inflammatory response and cell toxicity; (2) rAAV has an extremely broad range of host. In recent years, rAAV has been successfully transfected into brain, liver, lung, muscle, vascular endothelial and other organs (Summerford et al., 1999). It can be transduced into mitotic cells and non-dividing cells as well (Qing et al., 1998); (3) it exists for a long time and stably expresses up to 1.5 years in the infected cells (Xiao et al., 1996); (4) the physical and chemical properties of AAV are stable (Buning et al., 2008). It can be purified easily without deactivation, and can express target proteins long-termly, stably and efficiently *in vivo*.

Therefore, we used the rAAV as the carrier of target gene NT-1 to construct the vectors, and examined whether it can express NT-1 protein stably in the ischemic brain. We further demonstrated whether overexpression of NT-1 proteins could promote the migration of neural stem cells from subventricular zone (SVZ) area.

## Materials and Methods

### AAV Production, Purification and Titration

pAAV-NT-1 or pAAV-IRES-hr-GFP, pAAV-RC and pAAV-Helper plasmid were transfected into AAV293 cells (Wuhan Boster Biological Engineering Co., Ltd.) by calcium phosphate precipitation method. After harvesting cells 48 h after incubation in 37°C, cells were lysed by four freeze and thaw cycles (alternating between dry ice-ethanol and 37°C water baths). The lysates are centrifuged at 10,000 g for 15 min to remove the cell debris. The AAV vector is purified by Cesium chloride (CsCl_2_) gradient centrifugation. The gradient is fractionated 0.5–1 ml/fraction. Run SDS-PAGE to identify the containing viral fractions. The AAV vector-containing fractions are pooled, put in dialysis cassettes and dialyzed against 1 L buffer containing 10 mM HEPES (pH 7.4) three times at 4°C. The dialysis buffer is changed every 2 h. After that, virus solutions are frozen in the −80°C refrigerator for further use. RT-PCR is used for determining the titer of the virus. The CT values obtained by RT-PCR were used as the longitudinal coordinates, and numerical value of the number of standard concentration plasmid copies was used as the horizontal coordinates, the standard curve was then established. The viral titer was measured by the CT value (Rohr et al., 2002). The titer of AAV-GFP is 1.4 × 10^12^/ml and the titer of AAV-NT-1 is 2.8 × 10^12^/ml. The concentration of the injection is 1.4 × 10^12^/ml.

### Stereotactic Injection in Mouse Brain Basal Ganglia

Animal procedures were carried out according to a protocol approved by the Institutional Animal Care and Use Committee of Shanghai Jiao Tong University, Shanghai, China. Adult male CD-1 mice weighing 25–30 g were anesthetized with Ketamine (100 mg/kg) and xylazine (10 mg/kg, Sigma, San Louis, MO, USA) intraperitoneally. A burr hole was drilled 2 mm lateral to sagittal suture and 1 mm posterior to coronal suture in the left hemisphere. A 10 μl syringe (WPIInc., Sarasota, FL, USA) was slowly inserted into the brain 3 mm under the Dura. A 2.5 μl of AAV or saline was injected stereotactically at a rate of 0.2 μl/min. After half amount of liquid was injected, the needle was slowly withdrawn to 2 mm under the Dura to finish the injection. Ten minutes after injection, the needle was withdrawn from the mice undergoing a course of 15 min. The bone hole was sealed and the wound was closed. After sufficient awakening from anesthesia, animals were put back into their cages for long-term recovery.

### Transient Middle Cerebral Artery Occlusion (tMCAO) Model

Seven days after stereotactic injection, animals were anesthetized with 1.5% isoflurane in 70/30 nitrogen/oxygen gas for MCAO. The procedure of transient middle cerebral artery occlusion (tMCAO) was performed as described previously with little modification (Yang et al., 1994), the body temperature was maintained at 37 ± 0.5°C during surgery. Briefly, after the isolation of common carotid artery (CCA), external and internal carotid artery (ECA, ICA), left middle cerebral artery (MCA) was occluded by a 6-0 nylon suture coated with silica gel. Sixty minutes later reperfusion was achieved by partially withdrawing the suture from ICA to CCA. The blood flow values of the brain surface were monitored using laser Doppler flowmetry (Moor Instruments, Axminster, Devon EX13 5HU, UK). The successful tMCAO model was confirmed as a more than 80% decline in the ipsilateral hemisphere surface blood flow after occlusion and back up to 80% of baseline after withdrawing the suture.

### Western Blot Analysis

An equal amount protein of brain sample was loaded on 10% resolving gel for electrophoresis. Subsequently, proteins were transferred onto a nitrocellulose membrane (Whatman Inc., Florham Park, NJ, USA). The membrane was placed in 0.1% TBST with 5% non-fat milk lasting 1 h for blocking non-specific binding, and then immuno-probed with primary antibodies at 4°C overnight. After being washed with TBST, the membrane were incubated with HRP-conjugated secondary antibodies for 1 h at room temperature and then reacted with an enhanced ECL substrate (Pierce, Rockford, IL, USA). The result of chemiluminescence was recorded by an imaging system (Bio-Rad, Hercules, CA, USA).

### Immunohistochemistry and Immunofluorescence

Immunohistochemistry was performed according to the protocol described previously (Fan et al., 2008). After blocking with 10% bovine serum albumin, brain sections were incubated with primary antibodies at the following dilutions: NT-1 (1:100; Santa Cruz biotechnology Inc., Santa Cruz, CA, USA), NeuN and GFAP (1:500; Millipore Inc., Billerica, MA, USA), GluT-1 (1:300; Thermo, Waltham, MA, USA), nestin (1:200; Abacm, Cambridge, England) overnight at 4°C. Sections were then incubated with biotinylated or fluorescence-conjugated secondary antibodies. We used DAB immunostaining method for counting nestin^+^ cells. Each of stainings had appropriate positive and negative controls. Photomicrographs were taken from a confocal microscope (Leica, Solms, Germany). The intensity of staining was determined by Image pro plus software (Media Cybernetics Inc., Bethesda, MD, USA). We counted all brown positive cells per field in the SVZ region in each group, and then compared the number of nestin^+^ cells per field between groups at the same magnification.

### Statistical Analysis

Parametric data in different groups were compared using a one-way analysis of variance (ANOVA) followed by Student-Newman-Keuls test. Data are presented as mean ± SD. A probability value of less than 5% was considered as statistically significant.

## Results

### Expression of Target Protein After AAV Stereotactic Injection

**Figure 1:**
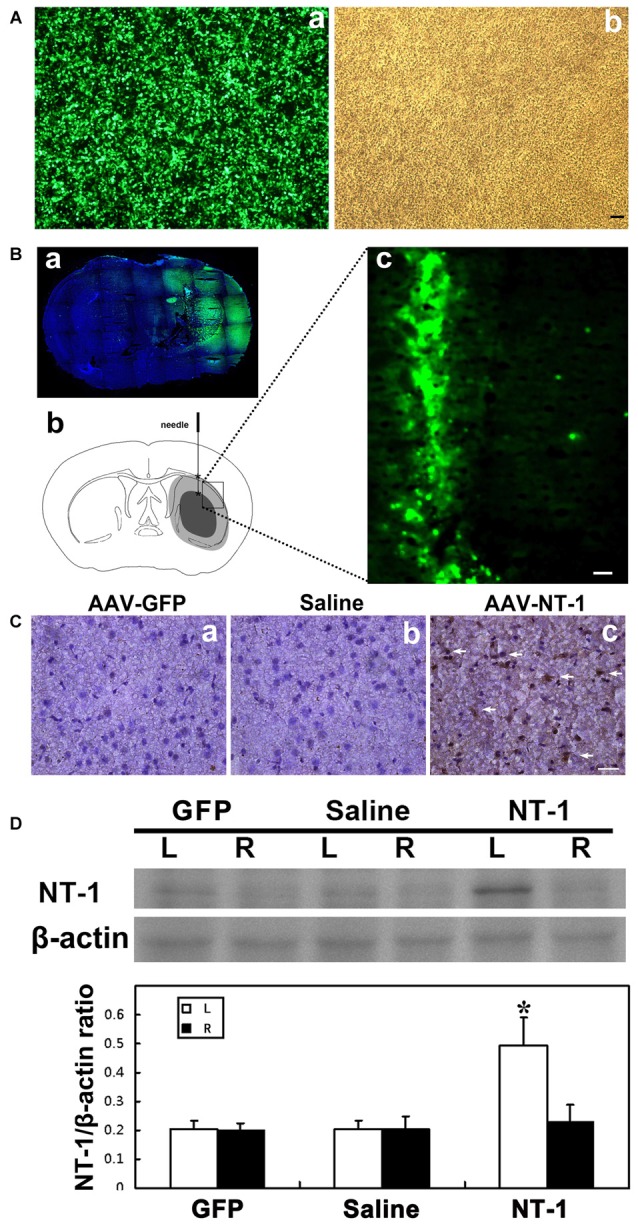
**Adeno-associated virus (AAV) successfully transduced into the brain. (A)** AAV293 cells in 40× fluorescence microscope **(a)** and light field **(b)** 48 h after AAV-GFP transfection. **(Ba)** Representative images of brain slices were shown by green fluorescence (GFP) counterstained with DAPI (blue) at 4 weeks after AAV-GFP injection. Green fluorescence was not seen in the contralateral side of the injection. **(b)** Pattern graph represents the brain coronal sections of mice. Asterisk is the virus injection site, which are in left hemisphere in two positions. The core area of ischemia is represented by black, and the surrounding area of ischemia is represented by gray. **(c)** The expression of green fluorescent protein in brain around the needle tracking 1 week after AAV-GFP injection. The scale bar is 50 μm. **(C)** Representative images of Netrin-1 (NT-1; brown) counterstained with DAPI (blue) in brain slice at 1 week after stereotactic injection. A large number of NT-1 positive cells were observed in the brain sections of AAV-NT-1 **(c)** transfected mice compared to the AAV-GFP **(a)** or saline **(b)** injected mice. The scale bar is 50 μm. The arrows indicated NT-1 positive cells. **(D)** Western-blot and its quantification for NT-1 in brain at 1 week after stereotactic injection: the upper blot bands were NT-1 protein strips with the molecular weight of 68 kDa and the lower blot bands were internal reference β-actin with the molecular weight of 42 kDa. L represents the left cerebral hemisphere; R represents the right cerebral hemisphere. The bar chart is the ratio of NT-1 protein vs. β-actin protein gray value. The expression of NT-1 in ipsilateral hemisphere of the AAV-NT-1 injection was significantly higher than that of the AAV-GFP and saline injection, and the contralateral hemisphere of the NT-1 injection as well. NT-1 represents AAV-NT-1 injected mice, Saline represents saline injected mice and GFP represents AAV-GFP injected mice. Data are shown as Mean ± SD. *N* = 5 in each group, *p < 0.001, left hemisphere in AAV-NT-1 injected group vs. other groups.

HEK293-FT was used to test the efficiency of AAV transfection; the result showed that almost 100% of cell presented GFP fluorescence (Figure 1A). Furthermore, we detect the expression of GFP at 1 week and 4 weeks after AAV-GFP injection into the mouse brain. Whole brain image showed that green fluorescent mainly expressed nearby needle track in the transduced hemisphere, not in contralateral hemisphere, and the expression lasted for 4 weeks after transfection (Figure 1B). The expression level of NT-1 is significantly increased in AAV-NT-1 mice brain compared to saline and AAV-GFP mice (Figures 1C,D). Meanwhile, western blotting results showed that the expression level of NT-1 is increasing only in transduced hemisphere, not in contralateral hemisphere.

### AAV-NT-1 Mainly Expressed in Neuron and Glial Cells After Transducing in Brain

**Figure 2:**
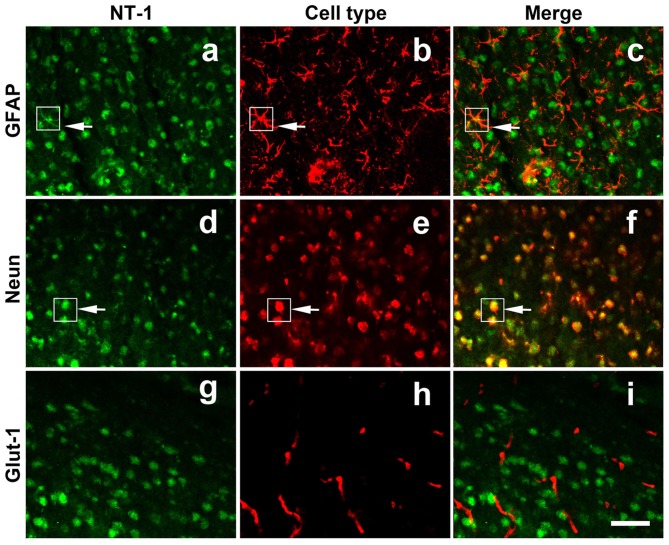
**NT-1 localization in the brain after AAV-NT-1 transfection.** Representative immunofluorescent images of brain slice at 1 week after AAV-NT-1 injection. Green fluorescence for NT-1 positive cells **(a,d,g)**; red fluorescence for GFAP **(b)**; Neun **(e)** or Glut-1 **(h)** positive cells. The arrows indicate the green and red co-localization cells **(c,f)**. NT-1 positive cells expressed GFAP **(c)** and Neun **(f)** but not Glut-1 **(i)**. The scale bar is 50 μm.

To clearly detect the identity of virus-infected cells *in vivo*, immunofluoresence staining was performed for NT-1, NeuN (neuron marker), GFAP (astrocyte maker) and Glut-1 (vascular endothelial cell marker) in frozen (thickness of 20 μm) sections of AAV-NT-1 transfected mice. The results showed that NT-1 was expressed in neurons and astrocytes, but not in vascular endothelial cells after transduction (Figure 2).

### Expression of NT-1 in Brain After tMCAO in AAV-NT-1 Treated Mice

**Figure 3:**
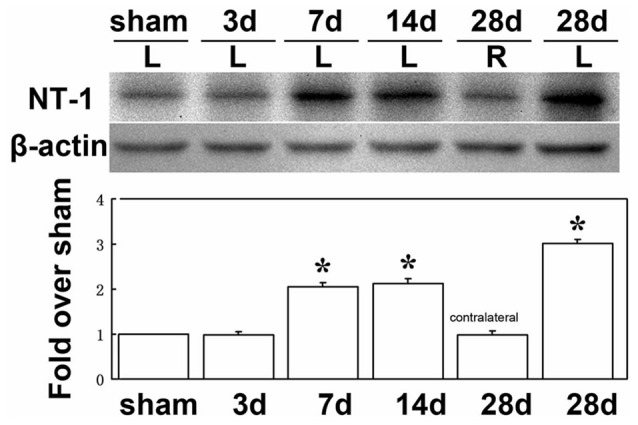
**The expression level of NT-1 in the brain after AAV-NT-1 transfection.** Western-blot and its quantification for the expression of NT-1 in brain. The upper blot bands were NT-1 protein strips with the molecular weight of 68 kDa and the lower blot bands were internal reference β-actin with the molecular weight of 42 kDa. Sham, 3, 7, 14 and 28 days, respectively represents sham injected mice and 3, 7, 14 and 28 days after AAV-NT-1 injected mice. L represents the left cerebral hemisphere; R represents the right cerebral hemisphere. The bar chart is the quantitative analysis of western-blot. Data are Mean ± SD. *N* = 5 in each group, *p < 0.001, left cerebral hemisphere in 7, 14 and 28 days after AAV-NT-1 injected mice vs. sham injected mice.

**Figure 4:**
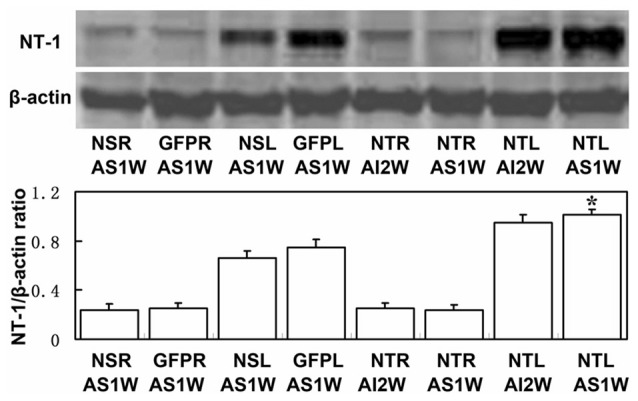
**The expression level of NT-1 in stereotactically injected mice after transient middle cerebral artery occlusion (tMCAO).** Western-blot and its quantification for the expression of NT-1.The upper blot bands were NT-1 protein strips with the molecular weight of 68 kDa and the lower blot bands were internal reference β-actin with the molecular weight of 42 kDa. NSR and NSL represent the right and left cerebral hemispheres of mice injected with saline. GFPR and GFPL represent the right and left cerebral hemispheres of mice injected with AAV-GFP. NTR and NTL represent the right and left cerebral hemispheres of mice injected with AAV-NT-1. AS1W represent the brain 1 week after tMCAO with AAV or saline injection 1 week later. AI2W represent the brain 2 weeks after AAV-NT-1 injection. The bar chart is the ratio of NT-1 protein to β-actin protein gray value. The expression of NT-1 in NTL/AS1W was significantly higher than that of NSL/AS1W and GFPL/AS1W, and the right brain as well. Data are presented as mean ± SD, *N* = 5 in each group, *p < 0.001, NTL/AS1W vs NSL/AS1W, GFPL/AS1W and the right hemisphere.

To clearly examine the change of NT-1 expression level with time after AAV-NT-1 transduction, we checked the expression level of NT-1 in brain in different time points after injection, the results showed that the expression of NT-1 was dramatically increased at 7 days after injection, and could keep high expression level till 28 days (Figure 3). To detect whether the expression of NT-1 will be affected in AAV-NT-1 transfected mouse after ischemia, western blotting was used to measure protein level in AAV-NT-1, AAV-GFP and saline stereotaxic injected mice 1 week after 60 min tMCAO left. We found that the expression level of NT-1 in the ipsilateral hemisphere of AAV-NT-1 group was higher after transient cerebral ischemia than that of AAV-GFP and saline group (Figure 4).

### Expression of Nestin in the Brain After Transient Cerebral Ischemia in AAV-NT-1 Mice

**Figure 5:**
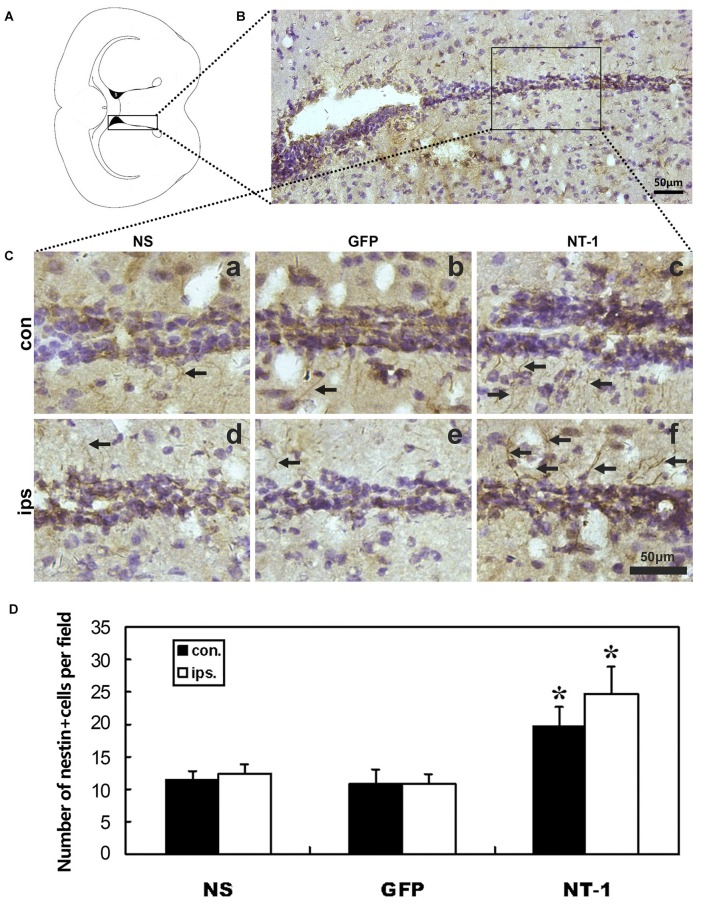
**Nestin expression in the region of subventricular zone (SVZ) in injected mice after tMCAO. (A)** Graphic illustration of a mouse brain coronal section we chose for nestin staining. **(B)** Nestin staining in SVZ region. **(C)** Represented images in AAV-NT-1, AAV-GFP and saline stereotaxic injected mice 1 week after tMCAO. Cells with brown color indicate nestin positive cells. The number of nestin positive cells are increased in AAV-NT-1 **(c,f)** transduced mice compared with saline **(a,d)** and AAV-GFP **(b,e)** injected mice. Scale bar is 50 μm. con means the contralateral side of injection; ips means the ipsilateral side of injection. The arrows indicated nestin positive cells. **(D)** Quantification of the average number of nestin positive cells per field. Data are presented as mean ± SD, *N* = 5 in each group, *p < 0.01, AAV-NT-1 treated mice vs. saline or AAV-GFP-treated mice.

In order to detect whether AAV-NT-1 transduction can affect the expression of nestin after tMCAO, immunohistochemical staining was performed in tMCAO mice 1 week after AAV-NT-1, AAV-GFP and saline stereotaxic injection. The result shows that the number of nestin^+^ cells was significantly increased in both hemispheres of SVZ region of AAV-NT-1 group compared to AAV-GFP and saline group after tMCAO. This result suggested that the migration of neural stem cells was improved in the SVZ in the AAV-NT-1 injected mice after tMCAO (Figure 5).

## Discussion

The early packaging method for AAV was always contaminated by adenovirus (Ad) or wild type generating with replication ability (Wang et al., 1998). Later, many attempts were made to improve the packing efficiency of rAAV vector (Tamayose et al., 1996; Gao et al., 1998). A variety of cells can be used as packaging cells, including 293 cells, Hela cells and so on. 293 cells with calcium phosphate transfection are commonly used for its high efficiency (Clark et al., 1995; Grimm et al., 1998). According to the method of Helper-Free System provided by Stratagene Company, we use a new auxiliary plasmid to produce AAV and avoid Ad contaminating. These plasmids containing VA, E2A and E4 gene of Ad genome, rep and cap gene of AAV, were transfected in 293 cells (AAV293 cells) with Ad5E1a and E1b gene. Helper plasmid provides an effective packaging system without pollution of Ad. Before the AAV293 cells were transfected with plasmid, IMDM medium was freshly changed to maintain the culture environment of AAV293 cells with optimum pH value and adequate nutrition (Xiao et al., 1998). The medium was changed with another medium containing 2% fetal bovine serum 6 h after transfection to limiting the excessive proliferation in the process of packaging virus. The harvest time of virus was controlled within 48–54 h after transfection, the exact time was determined by the cell morphology and transfection efficiency (especially for AAV-GFP). Harvest early, the number of virus was insufficient; harvest late, the virus was released from the cells reducing the harvesting amount of virus.

Cesium chloride density gradient centrifugation is the most commonly used method of separation and purification of all kinds of viruses. It is mainly based on the buoyant density characteristics of different viruses, which can separate other components from cell lysis solution. In this way, the virus can sometimes be obtained with a high purity. Most of the recombinant Ad used in clinical trials is purified by this method. However, they have obvious shortcomings in this method including time-consuming, difficult operation, poor reproducibility and cesium chloride toxicity. Therefore, with the development of research and application in gene therapy, the recombinant virus purified by this method could not be suitable for human application. Column chromatography, especially affinity chromatography, may be the main direction for purification of a large number of recombinant viral vectors used in clinical trials (Summerford and Samulski, 1999).

The method of measuring virus titer includes dolt-blot, RT-PCR and infecting cells, etc. Dolt-blot method is intuitive, but complicated in the operation and rough in quantitative aspect. The method of infecting cells can reflect the virus with infectious ability, but the infect situation is still different between the *in vivo* and *in vitro* experiment. So we used RT-PCR to quantify the virus titer and identify the virus. For AAV-GFP and AAV-NT-1, applied quantitative primer is directed against GFP and NT-1 specific gene fragments. Virus purity can be judged by melting curve. Scattered DNA chain was removed from virus solution with DNase I enzyme and the capsid of virus particles was decomposed by proteinase K before RT-PCR to exclude the gene fragments and empty viral capsids, then the actual number of intact virus particles were obtained (Rohr et al., 2002).

The brain was transduced with the virus by stereotactic injection. The expression of GFP was found in the vicinity of the needle trace 1 week after AAV-GFP transfection under fluorescent microscope. Immunohistochemical staining showed there were much more NT-1 positive brown cells around the needle trace in AAV-NT-1 group compared with AAV-GFP and saline groups. And the expression of NT-1 protein in the AAV-NT-1 group was significantly higher than that in the AAV-GFP and saline groups 1 week after injection. These results suggested that both AAV-GFP and AAV-NT-1 could be successfully transfected into mouse brains and expressed the target protein. At different time points after transduction, protein was extracted from the brain around needle tracks for quantitative analysis. Result showed that NT-1 expression was not significantly increased 3 days after transduction compared to the sham injected mice, while it was significantly increased compared with the sham injected mice in the subsequent 7–14 days and lasted up to 28 days. NT-1 expression in the ipsilateral hemisphere of AAV-NT-1 group was higher after transient cerebral ischemia than that of AAV-GFP and saline group. These results suggested that AAV could stably express the target protein *in vivo*, even after tMCAO (Davidoff et al., 2002). Since neurons and astrocytes were both inducible cells of AAV, AAV-NT-1 was mainly expressed in neurons and astrocytes (Fan et al., 2008).

NT-1 was proved to play an important role in peripheral nerve regeneration (Jaminet et al., 2013), facilitate axon outgrowth and induce cell migration (Bradford et al., 2009). Neural stem cells were found in SVZ region in the adults (Gonzalez-Perez and Quiñones-Hinojosa, 2012). NT-1 was proposed mainly as a long-range directional cue for many different types of neuronal precursors in the developing brain (Murase and Horwitz, 2002). NT-1 plays its function via combining receptors in various systems. As NT-1 receptors, DCC and UNC5H2 are broadly studied, but their functions in brain injury are still obscure. Both DCC and UNC5H2 are expressed in neural progenitor cells and involve in neuronal navigation during nervous system development (Fitzgerald et al., 2006; Wu et al., 2008). UNC5C, a transmembrane receptor of NT-1, plays a role in cognitive impairment during neurodegenerative disease (Sun et al., 2015). NT-1 binding with DCC displays both attraction and repelling effect, whereas NT-1 binding with UNC5H2 mainly shows repelling role (Mehlen and Furne, 2005). As a key factor, NT-1 up-regulated within the SVZ after injury and participated in local angiogenesis and progenitor cells migration. NT-1 not only directly promotes cells emigration but also contributes to local angiogenesis, which in turn indirectly facilitates progenitor cells emigration from the niche (Cayre et al., 2013). However, which receptor involved in progenitor cells migration needs further investigation.

Our previous study indicated that NT-1 promoted oligodendrocyte progenitor cells proliferation, differentiation and increased remyelination (He et al., 2013), which may relate to motor neuron functional recovery in some neurodegenerative disease like PD (Xu et al., 2012). In another experiment NT-1 was proved to promote endothelial cells proliferation and angiogenesis (Lu et al., 2012). Although other studies showed that nerve growth factor IB (Nur77), nuclear receptor related1 (Nurr1) and N-methyl-d-aspartate (NMDA) receptors also regulated neuroprotective signaling after neuronal injury of neurodegenerative disease (Tell and Hilgeroth, 2013; Wang et al., 2014; Wei et al., 2015), our study found that more nestin^+^ cells migrate from the SVZ to the infarct area in NT-1 group than that of the other two groups. These results suggested that different signaling pathway involved in different type of neuronal injury. NT-1 may promote the migration of neural stem cells to the infarct area for the neurological functional recovery, which might be one of the reasons for promoting oligodendrocyte progenitor cells and endothelial cells proliferation, the white matter repair and angiogenesis after tMCAO. NT-1/DCC signaling was proved to normally attract motor neurons closer to the floor plate (Kim et al., 2015). DCC could regulate appropriate precursor cell migration, axon guidance, and terminal arborization to improve the declined cognition (Xu B. et al., 2010). While in Schwann cells, NT-1-enhanced cell migration is mediated by activated p38 MAPK and PI3K-Akt signal cascades via receptor UNC5B (Lv et al., 2015). Therefore, how the NT-1 receptor is involved in recovery after stroke needs to be investigated in future.

## Author Contributions

HL: conception and design of the work, the interpretation of data for the work, drafting the work; XS, FW, GW, Yuncheng Wu, QW: the acquisition and analysis of data for the work; drafting the work; Yongting Wang, G-Y, ZZ: design of the work; the analysis of data for the work; revising the work critically for important intellectual content. All above authors give their final approval of the version to be published and agree to be accountable for all aspects of the work in ensuring that questions related to the accuracy or integrity of any part of the work are appropriately investigated and resolved.

## Funding

This work was supported by the National Key Research and Development Program of China (2016YFC1300600 G-Y), National Natural Science Foundation of China (81200898 HL; 81471178 G-Y; 81371305 YW), KC Wong foundation (G-Y) and the Science and Technology Commission of Shanghai Municipality (13ZR1422600 ZZ).

## Conflict of Interest Statement

The authors declare that the research was conducted in the absence of any commercial or financial relationships that could be construed as a potential conflict of interest.
